# Long-term peanut cultivation alters soil organic C stocks and aggregate stability: insights from coupled iron oxide, microbial, and lignin phenol analyses

**DOI:** 10.3389/fmicb.2026.1971411

**Published:** 2026-10-09

**Authors:** Yingru Liu, Shunli Cui, Shengchun Zhou, Haixin Wang, Yibo Wang, Puxiang Shi, Lifeng Liu

**Affiliations:** 1 College of Agronomy, Hebei Agricultural University/North China Key Laboratory for Crop Germplasm Resources, Ministry of Education/State Key Laboratory of North China Crop Improvement and Regulation, Baoding, China; 2 Liaoning Research Institute of Sandy Land Control and Utilization, Fuxin, China; 3 College of Forestry, Hebei Agricultural University, Baoding, China

**Keywords:** Iron oxides, long-term peanut cultivation, microbial- and plant-derived C, microbial attributes, soil organic C dynamics

## Abstract

**Introduction:**

The microbial and mineral C pumps play crucial roles in soil organic C (SOC) stabilization via complicated organo-mineral interactions. Yet, how both of them regulate SOC dynamics during peanut cultivation remains elusive.

**Methods:**

The surface soils from five peanut fields of varying ages (0-, 2-, 5-, 10-, and 20-yr.) were selected to elucidate the temporal variations in SOC dynamics and its potential mineral-microbial synergistic regulation patterns, based on PLFAs, eco-enzymatic activity, amino sugars, lignin phenols and Fe oxides analysis, etc.

**Results:**

With increasing peanut cultivation ages, SOC, labile C pools, microbial biomass, hydrolase activities, plant‑ and microbial‑derived C (PC and MRC), all exhibited unimodal patterns, peaking at 2-yr. peanut field before subsequently declining. Furthermore, the decreased Cy/Pre (i.e., the ratio of cyclopropyl PLFAs to their monoenoic precursor) and elevated fungi/bacteria values in older peanut fields indicated intensified bacterial physiological stress and a community shift favoring fungal dominance in these fields. Long-term peanut cultivation (i.e., 10 and 20-yr.) decreased SOC lability, enhanced aggregate stability, and altered SOC origins (i.e., enlarged PC but reduced MRC contribution to SOC). As peanut cultivation ages increased, the aggravated soil acidification (evidenced by a decline in pH from 6.10 to 4.57) concurrently enhanced the activation and complexation of Fe oxides, which was attributed to elevated levels of reactive Fe oxides (i.e., Feo and Fep). This transition altered the Fe–C coupling modes from ‘weak’ adsorption-driven (C:Fe molar ratios < 1) in 0- and 2-yr. peanut fields, to ‘strong’ complexation (1 to 6) in the, 5-, 10-, and 20-yr. peanut fields. The PLS-PM and Mantel test further suggested that intensified soil acidification promote reactive Fe oxides accumulation, while labile C pools and microbial biomass positively affect the accumulation of PC and MRC, respectively. Notably, reactive Fe oxides play a significant role only in MRC, rather than in PC accumulation.

**Discussion:**

Overall, these results elucidate the coupled mineral‑microbial influences on SOC dynamics over the peanut cultivation chronosequence, thereby enhancing our understanding of SOC sequestration processes in China’s peanut-producing regions.

## 1 Introduction

The peanut (*Arachis hypogaea* L.) represents a pivotal oilseed and cash crop within China, playing an indispensable role in ensuring the security of the national edible oil supply and augmenting farmers’ incomes (Chen et al., 2025a). Nevertheless, prolonged continuous monocropping has exacerbated issues such as soil acidification, nutrient imbalance, and alterations in microbial community structure within peanut cultivation regions, thereby posing a significant threat to the sustainable utilization of land resources (Yu et al., 2024). Among various soil properties, soil organic carbon (SOC) serves as a crucial indicator of soil quality and agroecosystem health, playing an essential role in ensuring agricultural sustainability and mitigating climate change (Chen et al., 2024). Consequently, a systematic elucidation of the temporal patterns of SOC stocks, their stability, and the underlying driving factors in the context of long-term peanut cultivation has emerged as a critical scientific issue for assessing soil degradation risks and developing strategies for fertility improvement (Sun et al., 2025). However, existing knowledge concerning the dynamics of SOC and its potential regulatory patterns during extended periods of peanut cropping is notably limited, necessitating comprehensive investigation.

Fundamentally, SOC is not a uniform pool and originates from plant residues via a series of intricate decomposition pathways, representing a continuum from fresh plant debris to microbially processed C (Li et al., 2020). A prevailing view holds that SOC buildup is fundamentally governed by the synchronous stabilization of organic C derived from plant and microbial sources (Chen et al., 2025b). Previous studies revealed that plant residues are widely accepted as the dominant contributors to the preservation of SOC pools; nevertheless, more and more evidence recognizes that microbial residues likewise contribute a substantial stable C pool (more than 50%) to SOC through mineral protection (He et al., 2025; Lei et al., 2026). Recently, it has been widely adopted that microbial- and plant-derived C can be precisely quantified by two biomarkers, i.e., amino sugars and lignin phenols, respectively (Qin et al., 2024; Wu et al., 2025). By using these technologies, Zhao et al. (2024) indicated that distinguishing dynamics of these two C sources is important for clarifying the pathways of SOC pool formation. Nevertheless, the current understanding of this information within peanut systems is limited, highlighting the need for a comprehensive investigation into the temporal dynamics of these C resources during peanut cultivation.

Emerging evidence and modeling have indicated that the accumulation of SOC, including microbial- and plant-derived C, in agroecosystems is fundamentally regulated by the dynamic equilibrium between its formation and subsequent mineralization loss (Li et al., 2024; Tang et al., 2024). This dynamic balance was closely associated with a series of complex interactions among physical, chemical, and microbial factors (Caruso et al., 2018; Liang and Zhu, 2021). Notably, microbes, which are sensitive to environmental and edaphic changes, play an important role in soil C cycling directly through their catabolic and anabolic functions (Zhang Y. et al., 2025). As discussed in the ‘microbial C pump (MCP)’ theory, exogenous C resources entering the soil undergo transformation through a dual microbial regulation process, which involves *in vitro* modification, i.e., microbial catabolism (depolymerization and respiration) by secreting exo-enzymes, as well as *in vivo* turnover, i.e., microbial anabolism for the synthesis of microbial biomass and necromass (Zhang S. et al., 2026). Through these processes, exogenous C is transformed into microbial metabolites and necromass, which was closely linked with microbial growth and decay, ultimately reinforcing SOC stabilization (Liang and Zhu, 2021). Recently, researchers have proposed the ‘mineral C pump (MnCP)’ theory, suggesting that mineral attributes, particularly iron oxides, play a crucial role in converting plant- and microbial-derived C into more stable forms through mineral-mediated abiotic processes in agroecosystems (Wang et al., 2026). According to Chen et al. (2025b), microbial necromass and a portion of plant-derived C can be stabilized in the soil by the MnCP through complex mineral-organic interactions, e.g., adsorption, aggregation, and polymerization, etc. These studies support the advancement of an innovative framework that integrates MCP and MnCP, wherein microbial turnover plays a crucial role in transforming exogenous C into microbial necromass, which is subsequently stabilized by mineral phases over prolonged timescales (Li et al., 2025). Current studies suggested that MCP and MnCP contribute unequally to SOC stocks in different ecosystems and that there are large differences in their contributions to C dynamics (Tang et al., 2025; Yan et al., 2026). Besides, the impact of peanut cultivation ages on SOC dynamics, along with the underlying patterns driven by MCP and MnCP theories, remains largely unexplored. This knowledge gap poses a challenge to the sustainable development of peanut industry in China, particularly in terms of SOC sequestration.

China accounts for 15% of the global peanut harvested area yet contributes 34% of world production, with over 90% of its national harvested area (92%) and output (94%) concentrated in four major regions, i.e., Northeast China, the North China Plain, the Yangtze River Valley, and Southeast China (Food and Agriculture Organization, 2024; National Bureau of Statistics of China, 2025). Liaoning Province serves as a major peanut production base in Northeast China, encompassing numerous peanut fields with diverse cultivation histories, which provides an optimal platform for investigating soil evolution under prolonged cultivation through a space-for-time substitution approach (Guo et al., 2021). In this study, we selected peanut fields with varying cultivation ages (0-, 2-, 5-, 10-, and 20-yr.) in this region. By systematically assessing aggregate stability, Fe oxides, microbial attributes, alongside biomarkers such as lignin phenols and amino sugars, etc., our objective is to elucidate the evolutionary patterns of SOC dynamics under long-term peanut cropping, and to uncover the synergistic patterns driven by MCP and MnCP (i.e., microbial traits and Fe oxide characteristics) that influence this process. The findings are anticipated to enhance our comprehension of SOC cycling and stabilization in cropland soils subjected to extended cultivation, through an innovative lens focusing on microbe–mineral interactions.

## 2 Materials and methods

### 2.1 Site description and experimental design

The peanut planting base, encompassing ca. 120 hectares, in Zhangwu County (42°53′N, 121°68′E, 223 m above sea level; Supplementary Figure S1) located in Fuxin City, Liaoning Province, China, was considered as the research site in this study. This region is a typical peanut producing area in Northeast China and is highly responsive to management, making it an ideal site for studying long term cultivation effects. Besides, this region has a semiarid continental monsoon climate, with mean annual precipitation and evaporation of 450–550 mm and 1,200–1,450 mm, respectively, along with mean annual temperature of 5.7 °C. Based on the FAO classification system, the soils at the study site are classified as aeolian sandy soil. Historical land-use records reveal that the area remained abandoned for over 20 years prior to the initiation of the experiment.

In contemporary research, the spatial–temporal substitution method is widely acknowledged as a reliable approach for assessing and detecting the dynamics of soil evolution processes over long-term scales (Guo et al., 2021). Since 2002, the unique edaphic and climatic conditions of this region have facilitated the widespread cultivation of the peanut (variety ‘Qinghua 6’). This extensive planting has resulted in the presence of numerous peanut fields of varying ages, providing an ideal opportunity to investigate the temporal changes in soil properties. Herein, peanut fields of different planting ages (2, 5, 10, and 20 years, abbr. as 2-, 5-, 10-, and 20-yr.) were selected as sampling sites within the study area; meanwhile, an adjacent wasteland (0-yr.) was chosen as a reference for comparative analysis. These planting ages were selected to represent different stages of soil evolution following peanut establishment. The 0-yr. site represented the reference condition before cultivation, the 2- and 5-yr. fields represented early cultivation stages, whereas the 10- and 20-yr. fields represented long-term continuous cultivation stages characterized by cumulative effects of agricultural management and soil environmental changes. A completely randomized design with three independent replicate plots per age-class was applied to each peanut field, resulting in a total of 15 plots, each covering ca. 225–250 m^2^. These fields were located in close proximity (less than 10 km apart), owned the same parent material, and were subjected to largely uniform agronomic practices under the management of the same agricultural cooperative. Due to their close spatial proximity and similar geomorphological characteristics, the trial plots exhibit broadly comparable climatic and edaphic conditions. Peanuts were cultivated using a single-seed, double-row ridge planting system. The ridges were spaced 60 cm apart, comprising a narrow row of 10 cm within each ridge and a wider row of 50 cm between adjacent ridges. The spacing between individual plants was maintained at 10 cm. These peanut fields were fertilized and managed based on the following regimen: Peanuts were sown in mid-May and harvested in late-October. Prior to sowing, a total of 750 kg ha^−1^ of compound fertilizer (N-P_2_O_5_-K_2_O: 15–15-15) was applied to the field, and the land was prepared using rotary tillage, performed once or twice. Abamectin and fludioxonil were employed for the management of insect pests and leaf spot disease. To mitigate drought stress, irrigation was performed 3 to 5 times during the entire growth period, contingent upon precipitation levels.

### 2.2 Soil sampling and aggregate fractionation

In May 2026, soil samples were collected from the topsoil layer (0–20 cm) in each plot. Within each plot, five undisturbed subsamples, each approximately 1.0 kg, were randomly gathered and subsequently combined to form one composite soil sample. After eliminating visible debris and gravels, these samples were carefully sieved using an 8-mm mesh to separate soil clods while preserving soil structure. The samples were then placed in rigid plastic containers and promptly transported to the laboratory for further analysis. Specifically, aside from the soil samples used for aggregate fraction analysis, the remaining soil samples were divided into two portions. One portion was air-dried and sieved through a 0.15-mm mesh to determine physico-chemical properties, microbial residues, and lignin phenols, etc.; the other portion was stored at −80 °C for the analysis of microbial attributes (e.g., microbial community and exo-enzymes).

The water-stable aggregates in the present study were isolated by the optimal-moisture sieving method (Luan et al., 2022a). Fresh bulk soil samples, which had been passed through an 8-mm sieve, were manually separated into three aggregate-size classes (i.e., >2 mm, 2–0.25 mm, and <0.25 mm aggregates) using a nested set of two sieves (2-mm and 0.25-mm). The detailed procedure for isolation is provided in Mao et al. (2021). Subsequently, the three aggregate fractions were oven-dried at 60 °C until a constant weight was achieved, then cooled and weighed to calculate their percentage content.

### 2.3 Soil physico-chemical analysis

Soil pH was determined in a 1:2.5 (w/v) soil-to-water suspension using a pH electrode. Soil organic C (SOC) and total N (TN) were determined by the K_2_Cr_2_O_7_-volumetric method and the Semimicro-Kjeldahl method, respectively (Bremner, 1960). Soil ammonium-N (NH_4_^+^-N) and nitrate-N (NO_3_^−^-N) were extracted with 2 mol L^−1^ KCl solution and measured by a continuous flow auto analyzer (AA3, Seal Analytical, Germany) (Esala, 1995). Soil available P (AP) was extracted by 0.5 mol L^−1^ NaHCO_3_ solution and subsequently determined by the molybdenum blue colorimetric method. Soil available K (AK) was extracted by 1.0 mol L^−1^ ammonium acetate and then measured with a flame photometer.

### 2.4 SOC pools analysis

The dissolved organic C (DOC) was extracted using distilled water at a 1:10 (w/v) soil-to-water, and subsequently quantified with a TOC analyzer (Multi NC 3100, Analytic Jena, Germany) (Novara et al., 2014). The light-fraction organic C (LFOC) was isolated using the density flotation method employing a sodium iodide solution with a density of 1.8 g cm^−3^, following the procedure outlined by Murage et al. (2007). Microbial biomass C (MBC) was assessed via the chloroform fumigation-extraction method (Brookes et al., 1985).

Besides, four types of oxidizable organic C were quantified based on the modified Walkley-Black method (Chan et al., 2001). The specific experimental steps can be found in Luan et al. (2022a). Based on the oxidizability and lability of SOC, it was classified into four distinct C pools: (i) very labile C (C1), oxidized with 5 mL H_2_SO_4_; (ii) labile C (C2), the difference in C oxidized between 5 mL and 10 mL H_2_SO_4_; (iii) less labile C (C3), the difference in C oxidized between 10 mL and 20 mL H_2_SO_4_; (iv) non-labile C (C4), residual C after oxidization with 20 mL H_2_SO_4_ when compared with SOC.

### 2.5 The determination of microbial-associated attributes

The microbial community composition in soils was assessed using the phospholipid fatty acids (PLFAs) analysis, following the methodology outlined by Yuan et al. (2016). In summary, lipids were extracted from ca. 3 g of freeze-dried soil utilizing a solvent mixture comprising citrate buffer, chloroform, and methanol in a volumetric ratio of 0.8:1:2. The phospholipid fraction was isolated using a silicic acid column and subsequently underwent alkaline methanolysis to yield fatty acid methyl esters (FAMEs). Afterwards, quantitative analysis was conducted with a gas chromatograph (Agilent 7890A), employing methyl nonadecanoate fatty acid (19:0) as the internal standard. Individual PLFAs were categorized into several microbial groups, including fungi, bacteria, and actinomycetes, as detailed in Supplementary Table S1, and their concentrations were expressed in nmol g^−1^. Besides, the ratios of cyclopropyl (cy19:0, cy17:0) to their monoenoic precursor (16:1ω7c, 18:1ω7c) fatty acids (Cy/Pre) were also calculated to evaluate microbial (bacterial) physiological status under environmental stresses (Wang et al., 2018).

The activities of seven soil exo-enzymes, comprising five hydrolases and two oxidases (see Supplementary Table S2), were quantified employing a microplate fluorescence technique as described by Bell et al. (2013). The hydrolase activities were assessed using fluorogenic substrates (e.g., 4-methylumbelliferyl and 7-amino-4-methylcoumarin); meanwhile, the oxidase activities were evaluated using *L*-3,4-dihydroxyphenylalanine (*L*-DOPA) as the substrate. The detailed descriptions of the specific experimental reagents, instruments, and procedures are available in the protocols outlined by Bell et al. (2013). The hydrolase and oxidase activities were measured using a microplate reader (Synergy 2, BioTek, USA) with 365 nm excitation and 450 nm emission filters or 450 nm absorbance, respectively. Enzyme activities were expressed in units of nmol g^−1^ soil h^−1^.

### 2.6 Amino sugars and lignin phenols analyses

The amino sugars (i.e., muramic acid, MurA; galactosamine, GalN; glucosamine, GluN) were analyzed following the methodology outlined by Zhang and Amelung (1996). The detailed experimental procedures are detailed in Guan et al. (2018), encompassing extraction (i.e., hydrolysis, filtration, and nitrogen evaporation) and derivatization to acetaldehyde derivatives. Subsequently, these amino sugars were quantified using inositol as an internal standard, which was introduced prior to hydrolysis. The quantification of MurA, GalN, and GluN in soil extracts was conducted utilizing an Agilent 6890A gas chromatograph (GC, Agilent Technologies, USA). The concentrations of amino sugars were reported in units of mg kg^−1^.

The lignin phenols were extracted and determined utilizing the alkaline cupric oxide (CuO) oxidation and gas chromatography technologies, respectively (Su et al., 2023). The detailed experimental procedures, including solvent extraction, alkaline hydrolysis, purification, and derivatization, were elaborated by Zhang J. J. et al. (2025). The lignin phenols (unit in mg kg^−1^) are classified into three types: (i) vanillyl (V) type, (ii) syringyl (S) type, and (iii) cinnamyl (C) type. Furthermore, the ratios of acid/aldehyde for V phenol (Ad/Al)v and S phenol (Ad/Al)s, along with the S/V and C/V ratios, were calculated to assess the degree of microbial oxidation and degradation of lignin, respectively (Qin et al., 2024).

### 2.7 Quantification of iron oxide fractions

Three distinct fractions of iron (Fe) oxide fractions, characterized by varying degrees of crystallinity, including free Fe oxides (Fed), amorphous Fe oxides (Feo), and organically complexed Fe oxides (Fep), were isolated via the selective extraction method (Duan et al., 2020). Specifically, the extraction of Fed, Feo, and Fep was achieved using dithionite–citrate–bicarbonate (DCB), 0.2 M ammonium oxalate–oxalic acid, and 0.1 M alkaline sodium pyrophosphate, respectively. The concentrations of these Fe oxides (expressed in g kg^−1^) in the extracts were subsequently determined by an ICP-OES Analyzer (iCAP 7,200, Thermo Scientific, USA). Besides, the activation index (AI = Feo/Fed) and complexation index (CI = Fep/Fed) were calculated to characterize the degree of activation degree and complexation of Fe oxides, respectively (Tang et al., 2025).

The Fe-bound C (Fe-OC) in soils was extracted and determined by the DCB extraction technique and reductive dissolution method, which selectively dissolves Fe oxides and releases their associated C into the extracts (Kramer and Chadwick, 2018). Each soil sample was separated into two subsamples, which were then extracted with DCB solution and NaCl solution (serving as a control), respectively. Specific descriptions of the experimental reagents, instruments, and procedures can be found in Duan et al. (2020). Then, the SOC in these subsamples was measured using an elemental analyzer (VELP EMA 502, Italy). The Fe-OC content was calculated as the difference in SOC between the DCB-treated and NaCl-treated subsamples.

### 2.8 Calculations

#### 2.8.1 Soil aggregate stability

The mean weight diameter (MWD) and geometric mean diameter (GMD) of soil aggregates, which were used for evaluating soil aggregate stability (Even and Cotrufo, 2024), were calculated based on the percentage content of >2 mm, 2–0.25 mm, and <0.25 mm aggregates and using the following equations:


$$\mathrm{MWD}=\sum_{i=1}^{3}(\mathrm{Xi}\times \mathrm{Wi})$$

$$\mathrm{GMD}=\mathrm{EXP}\sum_{i=1}^{3}(\mathrm{Wi}\times \ln(\mathrm{Xi}))$$

where Xi denotes the average diameter (mm) of aggregates, and Wi is the percentage content (%) of aggregates in the ith size.

#### 2.8.2 The lability of SOC

The lability index (LI) was commonly applied to assess SOC lability (Luan et al., 2022a). The specific calculation formula is as follows:


$$\mathrm{LI}=\frac{C1\times 3+C2\times 2+C3}{\mathrm{SOC}}$$

where C1, C2, and C3 denote very labile C, labile C, and less labile C, respectively.

#### 2.8.3 The geometric mean of the exo-enzyme activities

The geometric mean of the measured activities of hydrolase (GH) and oxidase (GOR), which is utilized to evaluate the overall status of microbial functions (Bell et al., 2013), was calculated as follows:


$$\mathrm{GH}=\sqrt[{5}]{\mathrm{\alpha G}\times \mathrm{\beta G}\times \mathrm{CBH}\times \mathrm{\beta X}\times \mathrm{NAG}}$$

$$\mathrm{GOR}=\sqrt[{2}]{\text{PHOs}\times \text{Perx}}$$

#### 2.8.4 The microbial- and plant-derived C

The biomarker-estimated fungal-, bacterial-, and microbial-derived C (FRC, BRC, and MRC; g kg^−1^) were calculated based on the contents of GluN and MurA (mg kg^−1^) (Hu et al., 2024):


$$\mathrm{FRC}=\frac{(\text{GluN}–1.16\times \text{MurA})\times 10.8}{1,000}$$

$$\mathrm{BRC}=\frac{\text{MurA}\times 31.3}{1,000}$$

where 10.8 and 31.3 were the conversion factors for fungal GluN to FRC and bacterial MurA to BRC, respectively. The MRC was calculated by aggregating the values of BRC and FRC.

Based on Qin et al. (2024) and Chen et al. (2025b), the values of V, S, and C were applied to calculate the biomarker-estimated plant-derived C (PC, g kg^−1^) using the follow formulas:


$$\mathrm{PC}=\frac{\left(\frac{V}{33.3\%}+\frac{S}{90\%}+C\right)}{22.5\%}$$

where 33.3 and 90% denote the release efficiencies for V- and S-type phenols by the oxidation of CuO, respectively. The 22.5% represents the average lignin phenol proportion reported for dominant terrestrial plant residues (Qin et al., 2024).

#### 2.8.5 The mode of Fe-C coupling

The C:Fe molar ratios can be utilized, to a certain degree, to assess the Fe-C coupling patterns in soils. These ratios were calculated using the formula provided by Zhang R. et al. (2026):


$$C:\mathrm{Fe}\;\text{molar ratios}=\frac{\mathrm{Fe}-\mathrm{OC}/\mathrm{Mc}}{M_{\mathrm{Fed}}/M_{\mathrm{Fe}}}$$

where M_Fed_, Mc, and M_Fe_ correspond to the concentration of Fed, the molar masses of C and Fe, respectively. When the C:Fe molar ratios are less than 1, between 1 and 6, or greater than 6, it indirectly suggests that the patterns of Fe-C interaction are primarily governed by adsorption, complexation, or co-precipitation, respectively (Wu et al., 2016).

### 2.9 Statistical analyses

One-way analysis of variance (ANOVA) was performed, supplemented by Duncan’s tests, to assess the impact of varying peanut planting ages on the indices outlined in the present study. Linear regression analysis was applied for exploring the relationships between MWD and several indices. The statistical analyses were executed using SPSS 22.0 software (SPSS Inc. Chicago, IL, USA). Principal component analysis (PCA) was conducted using CANOCO software (version 4.5) to discern differences in microbial communities across peanut fields of different ages.

The Mantel test and Spearman correlation analysis were performed utilizing the ‘ggcor’ and ‘dplyr’ packages to elucidate the relationships between MRC, PC, and edaphic variables (e.g., biotic variables, mineral indices, and abiotic parameters, a total of 14 parameters). Subsequently, partial least squares path models (PLS-PM) with goodness of fit > 0.6 was developed using the ‘plspm’ package to identify the major pathways of physico-chemical and microbial factors (e.g., pH, labile C, Fe oxides, and exo-enzymes, etc.) influence on PC and MRC. Furthermore, the direct and indirect impacts of edaphic variables on MRC and PC were investigated using the partial least squares (PLS) model with the packages ‘pls’. These analyses were performed using the R software (version 4.5.1).

## 3 Results

### 3.1 Soil aggregates distribution and stability

The marked differences were identified in the distribution of aggregate sizes within the surface soil layer (0–20 cm) across the five treatments in the study region, and the 0.25–2 mm aggregates constituted the largest proportion of all soil aggregates (35.0–42.0%), followed by the <0.25 mm (25.0–36.0%) and >2 mm aggregates (22.0–40.0%) (Figure 1a). Notably, the proportion of > 2 mm aggregates in the older peanut fields (i.e., 10- and 20-yr.) was significantly (*p* < 0.05) higher by 26.4–81.5% compared to those in 0- and 2-yr. fields; conversely, the proportions of 0.25–2 mm and < 0.25 mm aggregates exhibited inverse trends with the > 2 mm aggregates during peanut cultivation, i.e., 10- and 20-yr. < 2- and 0-yr. Furthermore, the values of MWD and GMD initially increased and then decreased during peanut cultivation, reaching their peaks in the 10-yr. peanut field (2.40 mm and 1.16 mm), which were significantly higher by 14.8–25.0% and 24.7–33.3%, respectively, than those in the younger peanut fields (i.e., 2- and 5-yr.) (Figure 1b).

**Figure 1:**
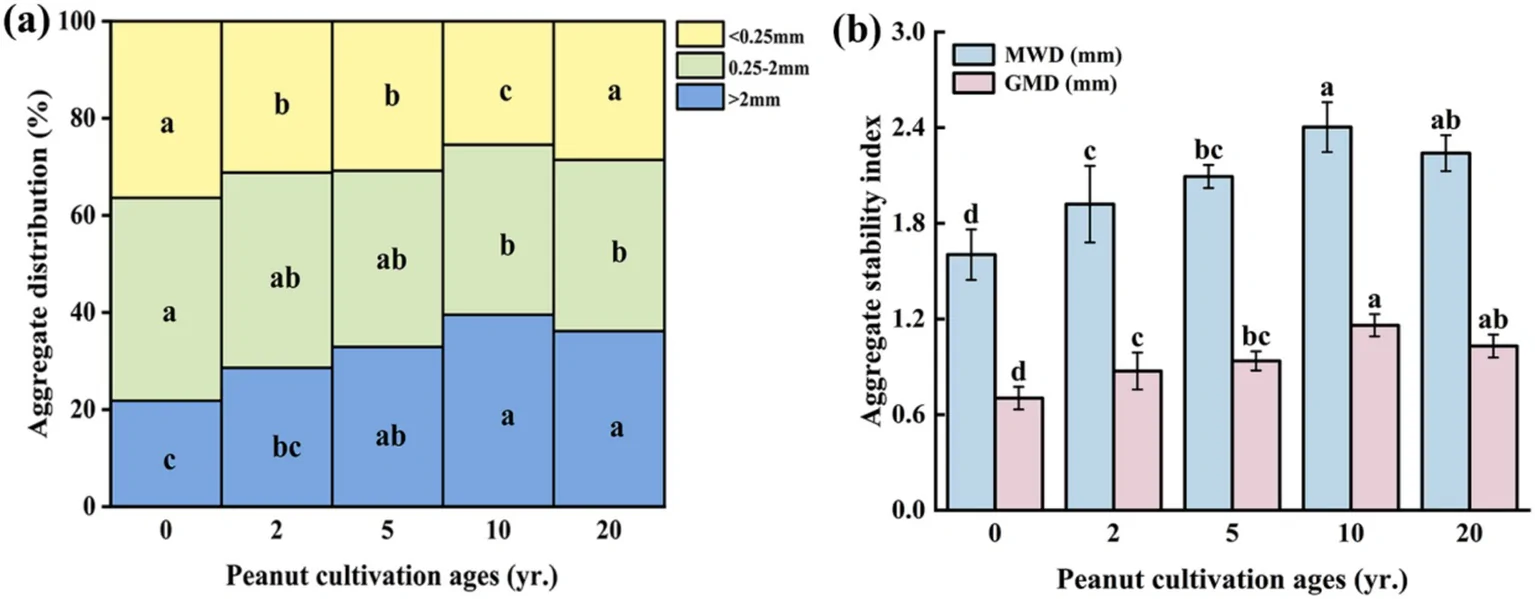
The soil aggregate size distribution **(a)** and aggregate stability indices **(b)** under different peanut cultivation ages. Different lowercase letters indicate significant differences (*p* < 0.05) among different treatments. MWD, mean weight diameter; GMD, geometric mean diameter.

### 3.2 Soil physicochemical variables

The physico-chemical properties (e.g., pH, SOC and TN, etc.) exhibited significant variability across the five different-aged peanut fields (Table 1). The SOC levels, which ranged from 8.15 to 12.38 g kg^−1^, showed a notable increase from the 0- to the 2-yr. peanut field, followed by a gradual decline from 2-yr. (12.38 g kg^−1^) to 20-yr. (8.15 g kg^−1^) peanut fields. Conversely, the contents of TN showed a progressive and significant increase with the duration of peanut cultivation, rising from the 0-yr. (0.63 g kg^−1^) to the 20-yr. (0.95 g kg^−1^) peanut field. The concurrent decrease in SOC and increase in TN during peanut cultivation resulted in a marked decline in the SOC/TN ratio, with values in the older peanut fields (8.6–11.4) being significantly lower by 24.5–52.0% compared to those in other fields (15.1–17.9). Additionally, the soils in the older peanut fields were characterized by higher levels of available N nutrients (i.e., NH_4_^+^-N and NO_3_^−^-N), and exhibited more acidic conditions (pH 4.57–5.12) compared to the younger peanut fields (pH 5.80–6.10).

**Table 1:** The physic-chemical properties of soil under different peanut cultivation ages.

Treatments	pH	SOC (g kg^−1^)	TN (g kg^−1^)	NH_4_^+^-N (mg kg^−1^)	NO_3_^−^-N (mg kg^−1^)	SOC/TN
0-yr.	6.10 ± 0.05a	11.09 ± 0.81b	0.63 ± 0.04e	1.78 ± 0.13d	16.4 ± 1.1c	17.7 ± 0.5a
2-yr.	5.85 ± 0.07b	12.38 ± 0.79a	0.69 ± 0.02d	2.13 ± 0.13c	19.0 ± 0.3c	17.9 ± 1.2a
5-yr.	5.80 ± 0.11b	11.51 ± 0.44ab	0.76 ± 0.02c	2.31 ± 0.09c	18.3 ± 1.9c	15.1 ± 0.8b
10-yr.	5.12 ± 0.08c	9.75 ± 0.41c	0.85 ± 0.04b	2.80 ± 0.11b	23.3 ± 2.3b	11.4 ± 0.7c
20-yr.	4.57 ± 0.13d	8.15 ± 0.52d	0.95 ± 0.05a	3.33 ± 0.26a	27.1 ± 2.6a	8.6 ± 0.6d

Data were shown as means values ± S.E. Different lowercase letters indicate statistically significant differences (*p* < 0.05) among different treatments. SOC, soil organic C; NH_4_^+^-N, ammonium-N; NO_3_^−^-N, nitrate-N.

### 3.3 SOC pools and its stability

The indices of SOC pools, e.g., DOC, LFOC, and oxidizable organic C in the present study, are listed in Tables 2, 3. In general, the labile C fractions (i.e., DOC, LFOC, C1, and C2), mirrored the trend of SOC across five peanut fields of varying ages, were highest in the 2-yr. peanut field, intermediate in the 0- and 5-yr. field, and lowest in the 10- and 20-yr. peanut fields; meanwhile, the contents of these C pools in the soils of the 2-yr. peanut field were significantly (*p* < 0.05) higher by 107.9–156.3%, 105.9–138.6%, 65–110.6%, and 38.5–101.7%, respectively, than those in the soils of the older peanut fields. Besides, the C3 contents were significantly higher by 30.4–72.9% in the younger peanut fields than in other treatments, whereas no significant differences were observed in the C4 contents across the five treatments (5.13–5.40 g kg^−1^, except for 20-yr.). Notably, the values of LI, an indicator for assessing SOC characteristics (Luan et al., 2022a), ranged from 0.92 to 1.23 and were 13.6–25.2% lower in the older peanut fields compared to other treatments.

**Table 2:** The contents of labile C, oxidizable organic C pools as well as SOC lability index under different peanut cultivation ages.

Treatments	Labile C pools		Oxidizable organic C pools (g kg^−1^)				LI
	DOC (mg kg^−1^)	LFOC (g kg^−1^)	C1	C2	C3	C4	
0-yr.	135.9 ± 13.2b	0.75 ± 0.05c	2.57 ± 0.23b	2.15 ± 0.22a	1.12 ± 0.26b	5.25 ± 0.38ab	1.18 ± 0.03a
2-yr.	179.8 ± 11.3a	1.05 ± 0.12a	2.97 ± 0.16a	2.34 ± 0.26a	1.66 ± 0.11a	5.40 ± 0.55a	1.23 ± 0.03a
5-yr.	159.9 ± 14.1a	0.89 ± 0.06b	2.6 ± 0.12ab	2.19 ± 0.18a	1.46 ± 0.18a	5.25 ± 0.09ab	1.19 ± 0.03a
10-yr.	86.5 ± 12.4c	0.51 ± 0.04d	1.80 ± 0.27c	1.69 ± 0.10b	1.12 ± 0.14b	5.13 ± 0.13ab	1.02 ± 0.04b
20-yr.	70.1 ± 3.6c	0.44 ± 0.02d	1.41 ± 0.10d	1.16 ± 0.09c	0.96 ± 0.09b	4.62 ± 0.50b	0.92 ± 0.05c

Data were shown as means values ± S.E. Different lowercase letters indicate statistically significant differences (*p* < 0.05) among different treatments. DOC, dissolved organic C; LFOC, light-fraction organic C; C1, very labile C; C2, labile C; C3, less labile C; C4, non-labile C; LI, lability index.

**Table 3:** The contents of soil microbial biomass, community composition, and associated ratios under different peanut cultivation ages.

Treatments	Total microbial biomass		Fungi (nmol g^−1^)	Bacteria (nmol g^−1^)	Actinomycetes (nmol g^−1^)	F/B	Cy/Pre
	MBC (mg·kg^−1^)	Total PLFAs (nmol g^−1^)					
0-yr.	151.2 ± 6.9c	15.1 ± 0.7b	2.30 ± 0.10b	8.93 ± 0.34b	0.69 ± 0.09ab	0.258 ± 0.003c	0.796 ± 0.011b
2-yr.	185.3 ± 11.5a	17.5 ± 1.0a	2.68 ± 0.13a	10.52 ± 0.66a	0.82 ± 0.09a	0.255 ± 0.008c	0.956 ± 0.023a
5-yr.	170.4 ± 7.1ab	16.3 ± 0.7ab	2.73 ± 0.18a	9.60 ± 0.58ab	0.79 ± 0.04a	0.285 ± 0.014b	0.943 ± 0.025a
10-yr.	155.2 ± 8.0bc	12.0 ± 0.4c	2.05 ± 0.09c	6.97 ± 0.41c	0.63 ± 0.10b	0.294 ± 0.013ab	0.819 ± 0.023b
20-yr.	138.7 ± 14.1c	11.0 ± 0.5c	1.89 ± 0.04c	6.24 ± 0.27c	0.58 ± 0.01b	0.303 ± 0.011a	0.735 ± 0.011c

Data were shown as means values ± S.E. Different lowercase letters indicate statistically significant differences (*p* < 0.05) among different treatments. MBC, microbial biomass C F/B, the fungi/bacteria ratio.

### 3.4 Soil microbial communities and its function

The microbial attributes (e.g., MBC, total PLFAs, and Cy/Pre, etc.) in the present study are shown in Table 3. Specifically, the total microbial biomass (as measured by MBC and total PLFAs), ranged from 138.7 to 185.3 mg kg^−1^ and 11.0 to 17.5 nmol g^−1^, respectively, initially increased with the age of peanut cultivation to reach the peaks at 2-yr. peanut field, and then sharply declined. Similarly, the trends observed for fungi, bacteria, and actinomycetes paralleled those of total PLFAs and MBC, specifically following the pattern of 2- and 5-yr. > 0-yr. > 10- and 20-yr. Besides, the ratios of F/B and Cy/Pre, which are utilized to assess microbial community structure and bacterial physiological stress (Moore-Kucera and Dick, 2008), respectively, exhibited inverse trends with increasing durations of peanut cultivation. Specifically, the F/B values were ordered as 0- and 2-yr. < 5-yr. < 10- and 20-yr., while the Cy/Pre values were significantly (*p* < 0.05) lower by 13.1–23.1% in the older peanut fields compared to the younger fields. The PCA analysis (Figure 2) further corroborated these findings, demonstrating that the duration of peanut cultivation reshaped microbial community structure, leading to clear distinctions among the five peanut fields of varying ages.

**Figure 2:**
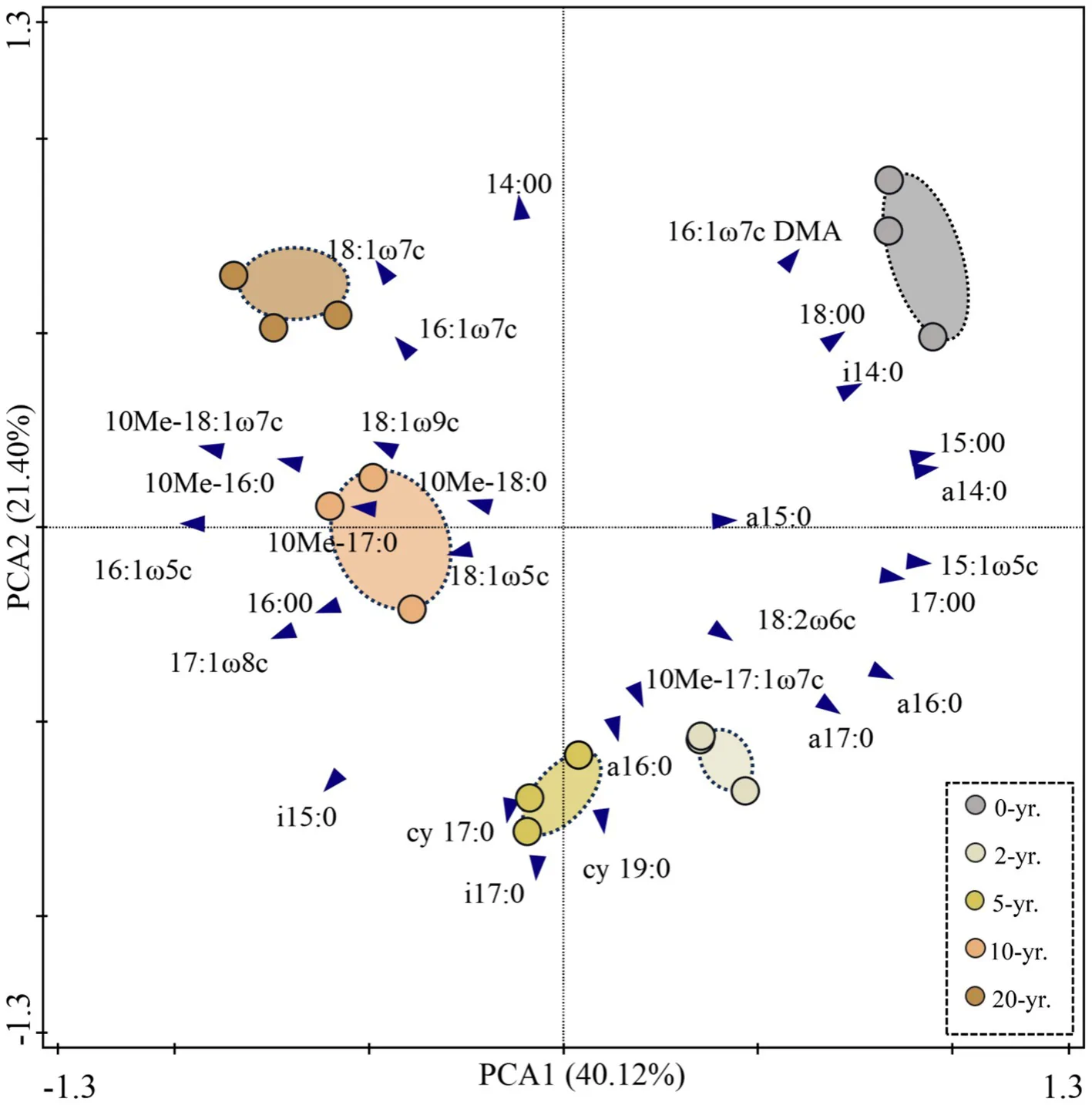
The principal component analysis (PCA) based on 32 individual PLFAs in the present study across different peanut cultivation ages.

The continuous cultivation of peanuts resulted in a significant change in exo-enzyme (i.e., hydrolases and oxidases) activities; nevertheless, the change trends of these two kinds of exo-enzymes were inconsistent among different-aged peanut fields (Table 4). The activities of hydrolase, including αG, βG, CBH, βX, NAG, and GH, first increased and subsequently decreased alongside peanut cultivation ages, and their values were consistently and significantly higher by 23.9–78.1%, 15.1–49.3%, 15.0–58.4%, 28.1–85.7%, 31.2–84.5%, and 22.4–70.1%, respectively, in the 2-yr. peanut field compared to other treatments. In contrast, the activities of oxidase, such as GOR, PHOs, and PerX, were generally lowest in the CK treatment, followed by the 2- and 5-yr. peanut fields, all of which were significantly (*p* < 0.05) lower than those observed in the 10- and 20-yr. peanut fields. Besides, the GH/GOR values were highest in the 2-yr. peanut field (2.36), intermediate in the 0- and 5-yr. peanut fields (1.99–2.00), and lowest in the older peanut fields (1.42–1.57).

**Table 4:** The exo-enzyme activities (nmol g^−1^ soil h^−1^) and the geometric mean of exo-enzyme activities in soils under different peanut cultivation ages.

Treatments	Hydrolase activities					Oxidase activities		The geometric mean of exo-enzyme activities		
	αG	βG	CBH	βX	NAG	PHOs	PerX	GH	GOR	GH/GOR
0-yr.	22.0 ± 1.3b	15.1 ± 1.7c	3.65 ± 0.42bc	6.28 ± 0.59b	9.96 ± 0.16b	3.51 ± 0.24d	3.65 ± 0.18b	9.5 ± 0.5c	3.58 ± 0.19c	2.00 ± 0.08b
2-yr.	30.1 ± 0.6a	22.1 ± 0.8a	4.69 ± 0.02a	8.56 ± 0.40a	14.56 ± 0.96a	3.99 ± 0.26c	3.88 ± 0.12ab	13.1 ± 0.3a	3.93 ± 0.19b	2.36 ± 0.05a
5-yr.	24.3 ± 1.8b	19.2 ± 0.6b	4.08 ± 0.27b	6.68 ± 0.15b	11.10 ± 1.32b	4.19 ± 0.14c	4.00 ± 0.16a	10.7 ± 0.4b	4.09 ± 0.15b	1.99 ± 0.01b
10-yr.	18.4 ± 2.5c	18.8 ± 1.2b	3.31 ± 0.20 cd	5.03 ± 0.44c	8.15 ± 0.64c	5.13 ± 0.13b	4.07 ± 0.11a	8.6 ± 0.4d	4.57 ± 0.12a	1.57 ± 0.05c
20-yr.	16.9 ± 0.5c	14.8 ± 0.8c	2.96 ± 0.37d	4.61 ± 0.31c	7.89 ± 0.75c	5.48 ± 0.09a	4.00 ± 0.10a	7.7 ± 0.2e	4.68 ± 0.10a	1.42 ± 0.01d

Data were shown as means values ± S.E. Different lowercase letters indicate statistically significant differences (*p* < 0.05) among different treatments. βG, β-Glucosidase; CBH, β-Cellobiosidase; βX, β-Xylosidase; αG, α-Glucosidase; NAG, N-acetyl-glucosaminidase; PHOs, phenol oxidase; PerX, peroxidase; GH, the geometric mean of hydrolase activities; GOR, the geometric mean of oxidase activities.

### 3.5 Soil microbial- and plant-derived C

The indices related to microbial-derived C (MRC), including fungal- and bacterial-derived C (FRC and BRC) are presented in Table 5. The concentrations of MRC exhibited a unimodal pattern throughout the cultivation chronosequence, increasing from 3.73 to 4.42 g kg^−1^ between the 0-yr. and 2-yr. fields, followed by a decline with continued cultivation. The contents of FRC and BRC, mirrored the trends observed in fungal and bacterial PLFAs (Table 3), displaying unimodal response patterns. These contents initially increased and subsequently decreased with the duration of peanut cultivation, reaching their maximum in the 2-yr. field (3.77 and 0.65 g kg^−1^, respectively). Notably, the values of MRC/SOC and FRC/BRC, which were used to appraise the microbial contribution to SOC and the composition of microbial residues, were significantly (*p* < 0.05) higher by 11.8–28.6% and 23.2–44.1%, respectively, in the older peanut fields compared to other treatments.

**Table 5:** The contents (g kg^−1^) of microbial-derived C and its associated ratios under different peanut cultivation ages.

Treatments	FRC	BRC	MRC	FRC/BRC	MRC/SOC (%)
0-yr.	3.18 ± 0.30bc	0.55 ± 0.03bc	3.73 ± 0.31bc	5.84 ± 0.58c	33.6 ± 2.0b
2-yr.	3.77 ± 0.23a	0.65 ± 0.04a	4.42 ± 0.27a	5.83 ± 0.12c	35.8 ± 1.5b
5-yr.	3.57 ± 0.21ab	0.61 ± 0.06ab	4.18 ± 0.27ab	5.85 ± 0.20c	36.3 ± 2.2b
10-yr.	3.47 ± 0.14abc	0.48 ± 0.03c	3.96 ± 0.17bc	7.21 ± 0.28b	40.6 ± 2.0a
20-yr.	3.14 ± 0.12c	0.37 ± 0.03d	3.51 ± 0.15c	8.40 ± 0.47a	43.2 ± 2.6a

Data were shown as means values ± S.E. Different lowercase letters indicate statistically significant differences (*p* < 0.05) among different treatments. FRC, fungal-derived C; BRC, bacterial-derived C. MRC, microbial-derived C.

The contents of plant-derived C, such as lignin phenols and PC, demonstrated age-dependent trends analogous to those observed in the patterns of MRC (Table 6). Specifically, soils from older peanut fields exhibited a reduction in plant-derived C (lignin phenols and PC) resources by 51.2–67.3 and 50–65.7%, respectively, in comparison to soils from younger peanut fields. Furthermore, the composition of lignin phenols was predominantly characterized by V-type phenols (60.1–66.0%), with their values being 2.33–9.82% higher in the older peanut fields relative to other treatments. The proportions of S-type phenols displayed an inverse trend compared to V-type phenols (i.e., 2- and 5-yr. < 10- and 20-yr.), while no significant differences were observed in the proportions of C-type phenols across the five treatments. These variations in the proportions of V-, S-, and C-type phenols resulted in lower C/V and S/V ratios in the older peanut fields compared to the younger fields. Besides, the values of (Ad/Al)v and (Ad/Al)s exhibited inverse age-dependent trends relative to the C/V and S/V ratios. Notably, the values of PC/SOC ranged from 13.9 to 26.6% during peanut cultivation and decreased significantly by 38.5–47.7% in the older peanut fields compared to those in other treatments.

**Table 6:** The lignin phenol contents, composition, and characteristics, as well as the contribution of plant-derived C to SOC under different peanut cultivation ages.

Treatments	Lignin phenol (mg kg^−1^)	VSC%			Lignin characteristics				PC (g kg^−1^)	PC/SOC (%)
		V	S	C	S/V	C/V	(Ad/Al)v	(Ad/Al)s		
0-yr.	281.3 ± 16.1b	60.1 ± 0.7b	26.1 ± 0.0a	13.8 ± 0.7a	0.44 ± 0.00a	0.23 ± 0.01a	0.38 ± 0.03c	0.42 ± 0.02a	2.79 ± 0.17b	25.2 ± 2.0a
2-yr.	331.3 ± 21.2a	60.2 ± 2.0b	25.6 ± 1.7a	14.2 ± 1.0a	0.43 ± 0.04a	0.24 ± 0.02a	0.40 ± 0.03bc	0.39 ± 0.04a	3.29 ± 0.21a	26.6 ± 0.6a
5-yr.	301.0 ± 4.2b	61.2 ± 1.8b	25.4 ± 0.1a	13.4 ± 1.7a	0.41 ± 0.01a	0.22 ± 0.03a	0.40 ± 0.02bc	0.42 ± 0.04a	3.02 ± 0.02b	26.2 ± 0.9a
10-yr.	146.8 ± 14.4c	64.5 ± 1.1a	22.7 ± 0.6b	12.9 ± 0.8a	0.35 ± 0.01b	0.20 ± 0.01a	0.45 ± 0.02ab	0.46 ± 0.04a	1.51 ± 0.14c	15.5 ± 0.9b
20-yr.	108.5 ± 10.9d	66.0 ± 1.8a	21.3 ± 0.7b	12.7 ± 1.1a	0.32 ± 0.02b	0.19 ± 0.02a	0.47 ± 0.06a	0.46 ± 0.04a	1.13 ± 0.12d	13.9 ± 0.9b

Data were shown as means values ± S.E. Different lowercase letters indicate statistically significant differences (*p* < 0.05) among different treatments. V, vanillyl type; S, syringyl type; C, cinnamyl type.

### 3.6 Soil reactive Fe forms and Fe-C coupling modes

Table 7 revealed that after a decade of peanut cultivation (i.e., 10- and 20-yr.), the Fed content was decreased by 0.3–11.1% compared with that in other treatments, while the contents of Feo and Fep were increased by 16.0–55.7% and 23.9–53.3%, respectively, resulting higher degree of activation and complexation (evidenced by elevated of AI and CI, *p* < 0.05) in the older peanut fields. Besides, a series of C-Fe coupling related indices (e.g., Fe-OC, fFe-OC, and C:Fe molar ratio) were analyzed to elucidate the mineral-driven patterns underlying the binding of Fe oxides to SOC. The Fe-OC contents and its proportion in SOC (fFe-OC) both exhibited a gradual increase with the age of peanut cultivation, being significantly higher by 11.6–28.9% and 33.1–75.2%, respectively, in the older peanut fields compared to other treatments. Notably, the C:Fe molar ratios across the five treatment followed the order: CK (0.90) < 2-yr. (0.99) < 5-yr. (1.13) < 10- and 20-yr. (1.28 and 1.29). These findings suggested a shift in the predominant Fe–C coupling patterns, with ‘adsorption’ (<1) being more prevalent in the younger peanut fields and ‘complexation’ (1–6) becoming dominant in the older fields.

**Table 7:** The contents and characteristics of Fe oxides, as well as the Fe-C coupling related indices under different peanut cultivation ages.

Treatments	The contents (g kg^−1^) of Fe oxides			The characteristics of Fe oxides		The Fe-C coupling modes		
	Fed	Feo	Fep	AI	CI	Fe-OC	fFe-OC (%)	C:Fe molar ratio
0-yr.	4.32 ± 0.09a	2.30 ± 0.15c	0.92 ± 0.03c	0.53 ± 0.05c	0.21 ± 0.01c	0.83 ± 0.01c	7.54 ± 0.55c	0.90 ± 0.01d
2-yr.	4.31 ± 0.31ab	2.41 ± 0.11c	0.94 ± 0.04c	0.56 ± 0.06c	0.22 ± 0.02c	0.92 ± 0.06b	7.45 ± 0.79c	0.99 ± 0.02c
5-yr.	3.93 ± 0.14bc	3.00 ± 0.09b	1.09 ± 0.03b	0.76 ± 0.05b	0.28 ± 0.01b	0.95 ± 0.02b	8.28 ± 0.23c	1.13 ± 0.05b
10-yr.	3.92 ± 0.17c	3.48 ± 0.09a	1.35 ± 0.05a	0.89 ± 0.05a	0.35 ± 0.02a	1.07 ± 0.05a	11.02 ± 0.33b	1.28 ± 0.08a
20-yr.	3.84 ± 0.17c	3.58 ± 0.10a	1.41 ± 0.04a	0.93 ± 0.04a	0.37 ± 0.03a	1.06 ± 0.02a	13.05 ± 0.94a	1.29 ± 0.04a

Data were shown as means values ± S.E. Different lowercase letters indicate statistically significant differences (*p* < 0.05) among different treatments. Fed, free Fe oxides; Feo, amorphous Fe oxides; Fep, organically complexed Fe oxides; AI, the activation index; CI, the complexation index; Fe-OC, Fe-bound C; fFe-OC, the proportion of Fe-OC to SOC.

### 3.7 Main explanatory variables of plant- and microbial-derived C accumulation dynamics

The Mantel test (Figure 3) was utilized to identify the key factors influencing the accumulation of MRC and PC. The analysis revealed that MRC exhibited strong correlations with biotic variables (i.e., total PLFAs, MBC, and GH; *p* < 0.01) as well as with abiotic parameters, including DOC (*p* < 0.05) and LFOC (*p* < 0.01). Similarly, PC also exhibited significant associations (*p* < 0.05) with DOC, LFOC, total PLFAs, and GH. To further explore the interactions among PC, MRC, and the array of mineral and microbial attributes across the cultivation age gradient, partial least squares path modeling (PLS-PM; GOF = 0.870, Fig. 74). The PLS-PM analysis revealed that peanut cultivation ages was negatively correlated with pH (path coefficient = −0.975^**^) and labile C pools (−0.802^**^), while being positively related to N fractions (0.966^**^). Consequently, pH was negatively associated with Fe oxides (−0.880^**^). Labile C pools, rather than pH and N fractions, were positively linked to microbial biomass (0.776^**^). Finally, microbial biomass (1.574^**^) and iron oxides (0.585^*^) were identified as important factors for positively regulating MRC. However, PC was solely associated with labile C pools (0.626^*^). Of note, the Partial Least Squares (PLS) model revealed that the cumulative impact of peanut cultivation age on both MRC and PC was negative, which was largely mediated indirectly via pH, N fractions, and labile C pools (Figures 4b,c).

**Figure 3:**
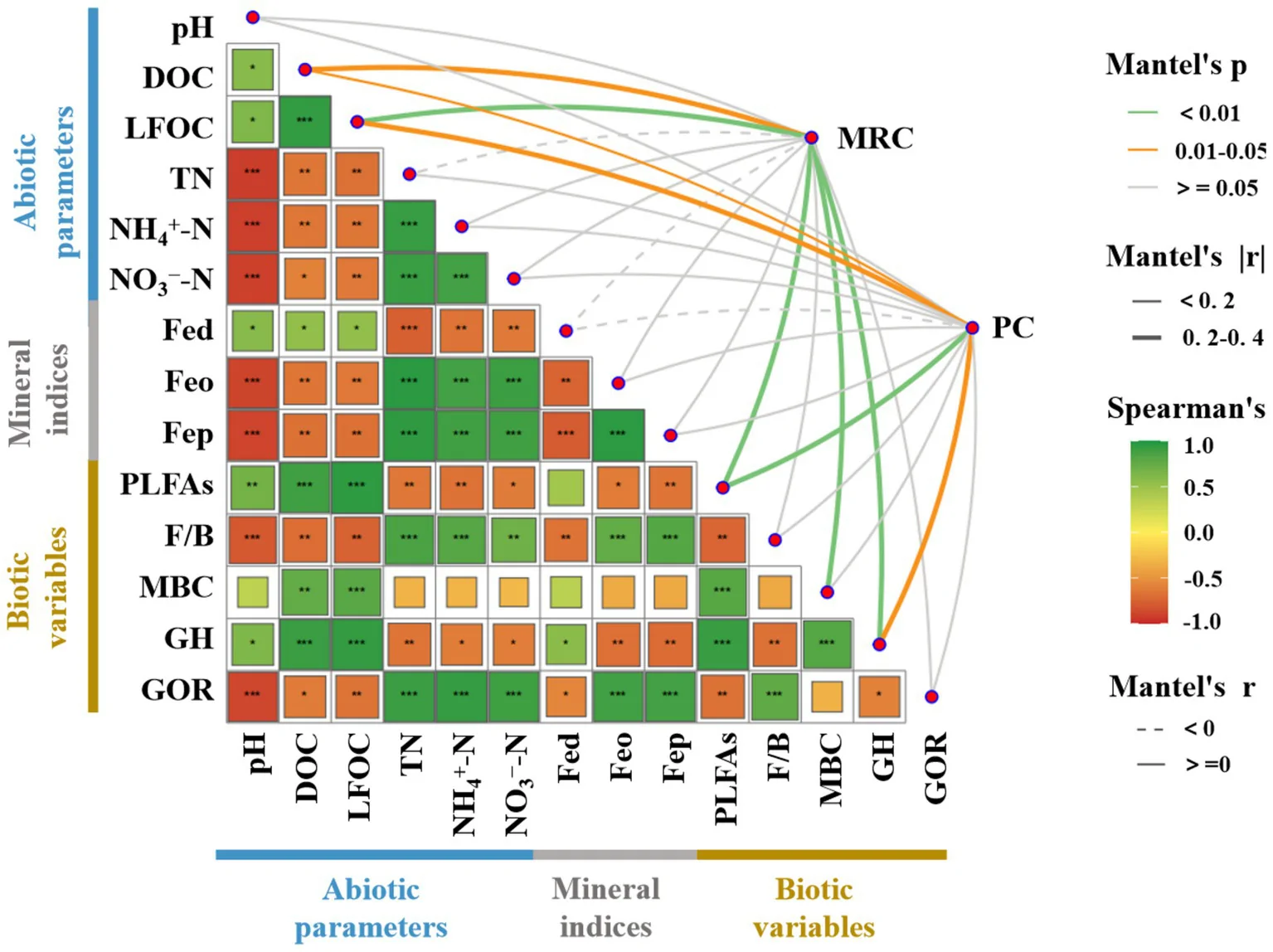
The results of the Mantel tests and Spearman correlation matrix illustrate the relationships between microbial- and plant-derived C and a series of edaphic parameters, e.g., abiotic, biotic, and mineral indices, along peanut cultivation processes. DOC, dissolved organic C; LFOC, light-fraction organic C; TN, total N; NH_4_^+^-N, ammonium-N; NO_3_^−^-N, nitrate-N; Fed, free Fe oxides; Feo, amorphous Fe oxides; Fep, organically complexed Fe oxides; MBC, microbial biomass C; F/B, the fungi/bacteria ratios; GH, the geometric mean of hydrolase activities; GOR, the geometric mean of oxidase activities.

**Figure 4:**
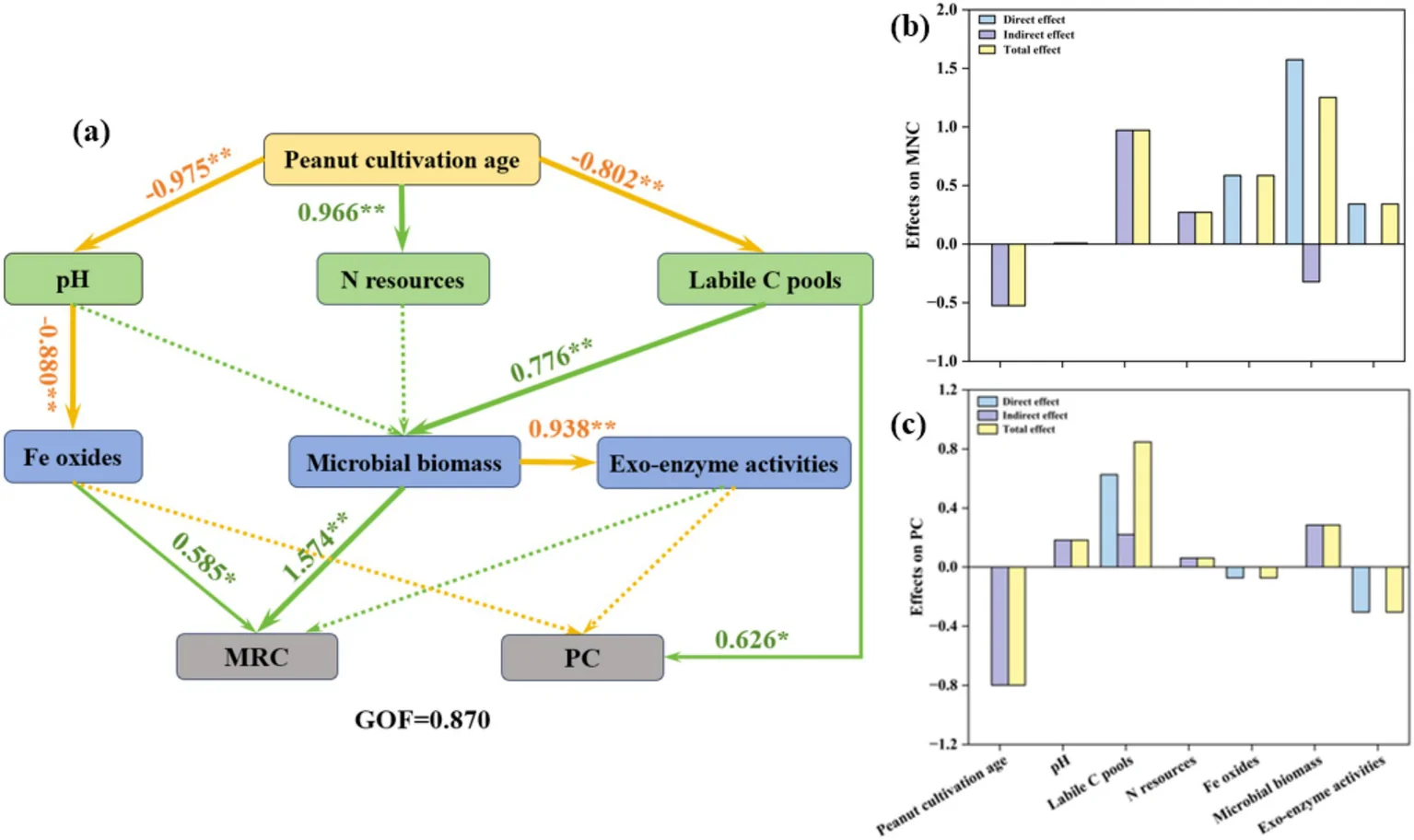
Partial least squares path model (PLS-PM) disentangling major pathways of environmental influences (i.e., soil biotic, abiotic and mineral indices) on plant- and microbial-derived C (PC and MRC) **(a)**, and their standardized direct and indirect effects derived from PLS-PM on MRC **(b)** and PC **(c)**. The arrows indicate the hypothesized direction of causation. The green or yellow solid arrows indicate significant positive or negative relationships, respectively. Dashed arrows indicate non-significant relationships from *a priori* model. The following variables were included: N resources (NH_4_^+^-N, NO_3_^−^-N, and TN); labile C pools (DOC, LFOC); microbial biomass (Total PLFAs, MBC, and F/B); iron oxides (Feo and Fep); exo-enzyme activities (GH and GOR). Asterisks indicate the statistical significance (^**^p < 0.01 and ^*^p < 0.05).

## 4 Discussion

### 4.1 Enhanced soil aggregate stability despite concomitant depletion of soil organic C under long-term peanut cultivation

This study demonstrates that the levels of SOC pools (e.g., LFOC and DOC, illustrated in Table 2) as well as nutrient resources (e.g., TN, NH_4_^+^-N, NO_3_^−^-N, presented in Table 1) exhibit significant divergence across five different-aged peanut fields. These variations are closely associated with the patterns of exogenous resource (e.g., fertilizers) inputs and their utilization by crops during peanut cultivation (Zhou et al., 2025). The older peanut fields were characterized by elevated levels of N resources, coupled with reduced C resource levels, constituting a ‘C-low vs. N-high’ pattern. This N-rich pattern likely arises from the accumulation of chemical N fertilizers and atmospheric N fixation facilitated by legume root nodules, jointly provide a large, readily available N subsidy to the soil (Hu et al., 2023; Salinas-Roco et al., 2024). The elevated SOC and labile C pools observed in the 2-yr. field can be attributed to the external C supply provided by root residues during the initial stages of peanut cultivation, which was adequate to sustain microbial growth and SOC accumulation. Nevertheless, with extended peanut cultivation, the imbalance between the marked cumulative depletion of C resources due to microbial decomposition and the inadequate C inputs (i.e., absence of organic fertilizer inputs) has led to reduced SOC contents in older peanut fields (Table 1) (Ahsan et al., 2024; Jiang et al., 2026). Notably, we observed that long-term (i.e., 10- and 20-yr.) peanut cultivation could decrease SOC lability, as evidenced by lower LI values (Table 2). This phenomenon can be attributed to the consumption of labile C, which could be related to the imbalance between soil C resources vs. microbial C demand (Zhang et al., 2024). In older peanut fields characterized by ‘poor’ C levels (see Table 2), microbes could only obtain the necessary energy to sustain their metabolic activities by degrading C resources (especially labile C1 and C2; Table 2). So, the limited input of organic fertilizers, which are predominantly composed of labile C, coupled with loss by microbial consumption, has collectively contributed to these results.

Interestingly, our study revealed that prolonged peanut cultivation significantly altered the composition of soil aggregates by increasing those >2 mm aggregates and reducing other aggregates, while also enhancing aggregate stability as measured by the elevated values of MWD and GMD, despite the depletion of SOC (Table 1). Prior research identified that SOC is a key cementing agent for soil aggregation and facilitate the formation of > 2 mm aggregates, which contradicted our findings, i.e., MWD and GMD increased in the elder peanut fields, whereas SOC and labile C pools declined (Figure 1 and Table 1). One plausible explanation appears to be linked to the ‘role transition’ of the cementing agent responsible for aggregation, transitioning from SOC to Fe oxides and Fe-OC (Huang et al., 2018). Bronick and Lal (2005) posited that the formation of soil aggregates is intricately connected to a specific process involving the creation of a cation bridge by multivalent metal cations (e.g., Fe oxides) bonding with organic compounds. Wu et al. (2016) also indicated that Fe oxides (i.e., Feo and Fep) are strongly associated with aggregate stability in zonal soils in South Central China, which aligned with the findings presented in Figure 5 (i.e., strong correlation between Feo, Fep, Fe-OC, and MWD). In essence, although the levels of SOC decreased in the older peanut fields, the increased presence of Feo, Fep, and organo–mineral complexes (i.e., Fe-OC formation) offers an alternative powerful cementing agent for soil aggregation in the current study. Notably, it was observed that increased aggregate stability did not correlate with higher SOC accumulation in the present study. While improved soil aggregation is known to enhance the physical protection of SOC (Even and Cotrufo, 2024), the overall SOC stocks are primarily governed by the equilibrium between C inputs and losses. In other words, under extended peanut cultivation, insufficient C inputs, coupled with continuous C removal and microbial utilization/mineralization, likely led to cumulative C losses surpassing C inputs. Consequently, SOC levels declined in the older fields despite the observed enhancement in aggregate stability.

**Figure 5:**
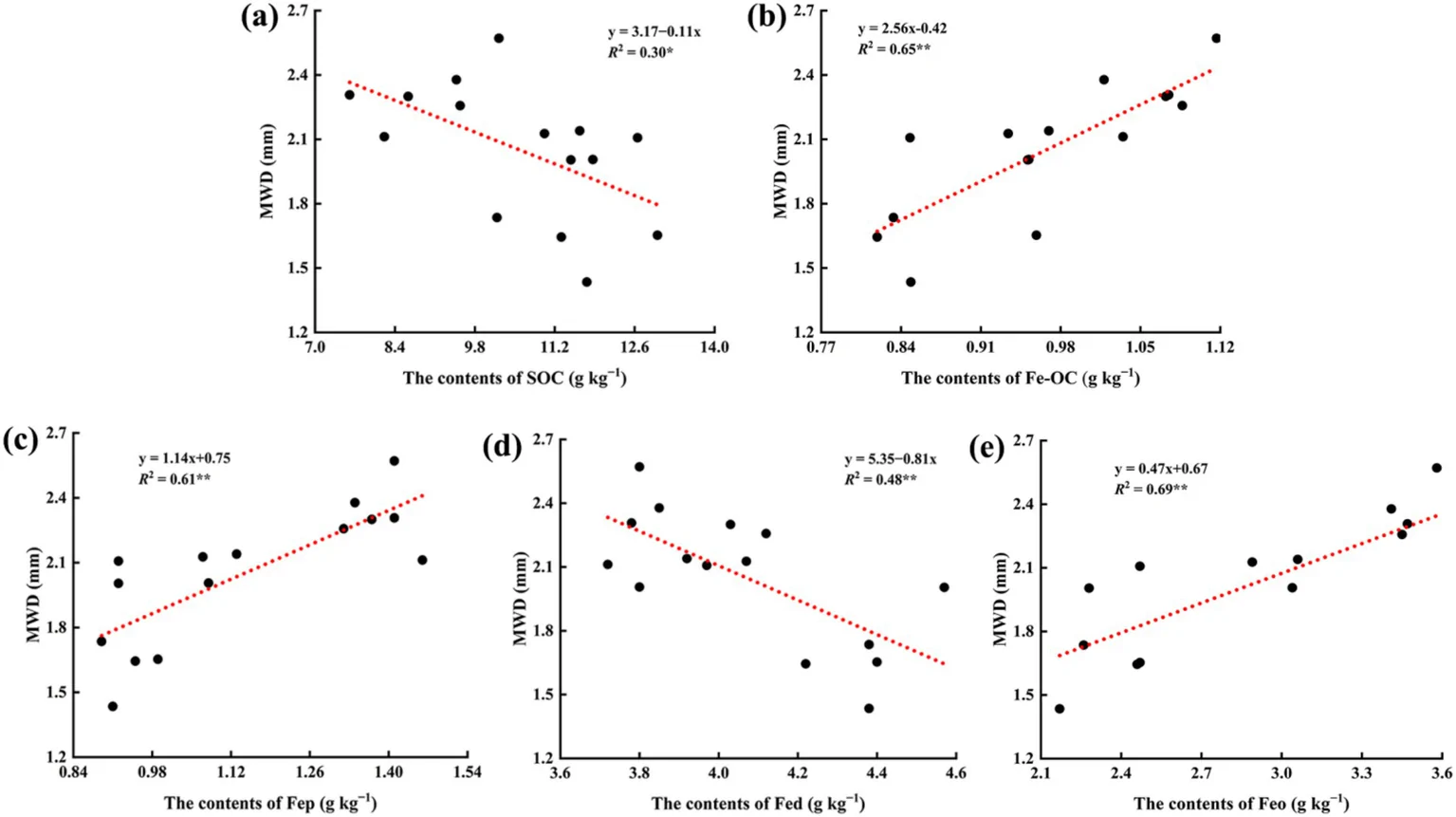
Relationships between MWD and the contents of SOC, Fe-OC, and iron oxides, i.e., SOC **(a)**, Fe-OC **(b)**, Fep **(c)**, Fed **(d)**, and Feo **(e)**. ^*^ and ^**^ indicate significant at *p* < 0.05 and *p* < 0.01, respectively. MWD, mean weight diameter; SOC, soil organic C; Fe-OC, Fe-bound C.

### 4.2 The soil acidification and poor C resources caused by prolonged peanut cultivation inhibits the growth and functions of microbes

Recent research has identified microbial attributes, such as microbial communities and exo-enzyme activities, are pivotal microbial drivers for SOC dynamics within agroecosystems (Xiao et al., 2025; Luan et al., 2022a). The results obtained herein (Tables 3, 4) corroborated the unimodal response of microbial biomass to varying durations of peanut cultivation, with peaks observed in 2-yr peanut field and declining thereafter (Table 3). This pattern aligns with prior finding (Upton et al., 2019) but contrasts with the result reported by (Yang et al., 2020). Such discrepancies can be attributed to differences in agricultural management practices, edaphic attributes, and environmental conditions across different study sites (Li et al., 2022). In the present study, the soils in the 2-yr. peanut fields offered sufficient ‘food’ for microbial proliferation (i.e., higher C and N resources; see Table 1), which were beneficial for increasing microbial biomass in these fields (Wang and Kuzyakov, 2024). Conversely, the soils of older peanut fields, which have experienced a prolonged absence of exogenous C (i.e., organic fertilizers) inputs, exhibit reduced C resources (Table 2). The reduction of SOC, particularly the labile C pools, likely represented the primary constraint on microbial growth in the older peanut fields, given that microbes rely directly on these C resources for their survival (Wang et al., 2023). Concurrently, the aggregated acidic soil conditions (pH < 5.04) may serve as an additional environmental stressor, further impeding microbial growth under prolonged peanut cultivation (Rothenberg et al., 2022). Consequently, the limited C resources in the older field, coupled with low pH values (Table 1), are likely to inhibit microbial growth in the current study. Notably, our findings indicated that the soils in the older peanut fields were more conducive to fungal growth than bacterial growth, as evidenced by the elevated F/B values (Table 3). This observation was closely associated with the microbial ‘instinct characteristics’ (Wang and Kuzyakov, 2024) and the variations in edaphic attributes across all treatments. Prior research has highlighted that soils with high acidity and ‘poor’ C resources tended to promote fungal growth over bacterial proliferation (Dai et al., 2017). Consequently, the soils in the older fields, characterized by low pH values (4.57–5.12) and limited labile C resources (Table 2), provide more favorable microsites for fungal growth compared to bacterial growth, resulting in higher F/B values in these soils. Additionally, this alteration in the F/B values partially elucidates the increased stress experienced by bacteria in these soils, as indicated by the decreased Cy/Pre values (Table 3) in these soils.

Our study underscores that long-term peanut cultivation led to a reduction in hydrolase activities and an increase in oxidase activities (Table 4), which are intricately associated with microbial growth patterns (Zhang S et al., 2025) and the exo-enzymatic substrate preferences (Luo et al., 2017). Sokol et al. (2022) have demonstrated that exo-enzymes, i.e., hydrolases and oxidases, are predominantly secreted by bacteria and fungi, respectively. Consequently, the diminished microbial biomass (particularly bacteria) in the older peanut fields (Table 3) was not conducive to hydrolase production. Notably, the observed decrease in fungal biomass seems to be contradictory to the increased oxidase activities in these soils. This paradox can be elucidated by the findings of Wang and Kuzyakov (2024), who indicated that fungi can enhance their adaptability to adverse environments by secreting more oxidases when soil resources are limited. Therefore, although the suboptimal edaphic conditions (i.e., limited C resources) in the older peanut fields were not favorable for fungal growth (Table 3), they may have stimulated fungi to secrete more oxidases as an adaptive response, thereby partially explaining the observed increase in oxidase activities in these soils in the present study.

### 4.3 The soil acidification caused by prolonged peanut cultivation changed Fe forms and their effects on SOC retention

Our findings corroborate that long-term peanut cultivation strongly elevates the levels of Feo and Fep, while concurrently reducing Fed levels, thereby enhancing the activation and complexation of Fe oxides in the older peanut fields (Table 7). These findings partially aligned with the findings obtained by Tang et al. (2025), who reported an increase in Fe oxides (e.g., Feo and Fep) with extended durations of tea cultivation in Jiangsu province, China. These observations can be attributed to the discrepancies in physicochemical characteristics (e.g., pH) with peanut cultivation processes in the present study (Vermeire et al., 2019). Yu et al. (2017) indicated that the prolonged application of chemical N-based fertilizers gradually releases more organic acids (e.g., tannic acid and humic acid, etc.) into soils. Besides, Salinas-Roco et al. (2024) revealed that biological N fixation by peanut rhizobia may also contribute to acidification through proton release during charge balance regulation and organic acid exudation from roots. These organic acids could exacerbate soil acidification and facilitate the dissolution of Fe mineral phases, thereby forming more reactive Fe oxides (e.g., Feo and Fep). The results presented in the PLS-PM analysis (see Figure 4) supported this, demonstrating that pH has a significant negative impact on Fe oxides.

Notably, the Fe-OC content and fFe-OC in the older peanut fields were significantly higher compared to other treatments, which can primarily be attributed to the enhanced Fe-C interaction modes that facilitate the formation of Fe-OC in these soils (Tang et al., 2025) and was partially supported by the results of Table 7 (i.e., higher values of AI and CI). Furthermore, Jeewani et al. (2021) revealed that the presence of poorly crystalline Fe oxides (i.e., Feo and Fep), known for their high reactivity and specific adsorption surfaces, plays an important role in the formation of Fe-OC. Therefore, the elevated levels of Feo and Fep, induced by low pH values in the older peanut fields, were advantageous for the production of Fe-OC and inhibited its decomposition by microbes. Besides, the data pertaining to C:Fe molar ratios (Table 7) in the present study indicated a transition in Fe-C coupling modes from weak surface ‘adsorption’ in the younger peanut fields to strong ‘complexation’ in the older fields (Wu et al., 2016). The increased acidic levels in the older peanut fields may facilitate the dissolution and transformation of Fe oxides by modifying their deprotonation processes, thereby enhancing Fe-C affinities and potentially leading to the formation of denser and stronger Fe-C ‘complexation’ (Tang et al., 2025). This intensified Fe-C ‘complexation’ in the older fields also accounts for the observed higher Fe-OC levels in these fields.

### 4.4 Shifting C sources from plant- to microbial-origins: a compositional transformation of SOC under prolonged peanut cultivation

Our findings demonstrated a notable alteration in the source of SOC during peanut cultivation ages. Both plant- and microbial-derived C contents exhibited unimodal distribution patterns, reaching their peak in 2-yr. peanut fields before declining with the increasing age of peanut cultivation (Tables 5, 6). This observation partially contradicted several findings from chronosequence studies conducted in forest and garden ecosystems (Luan et al., 2022b; Xu et al., 2026). The accumulation of microbial- and plant-derived C were closely associated with the balance between the formation and subsequent preservation of these C resources (Kallenbach et al., 2016; Hu et al., 2024). Exogenous C and microbial biomass are recognized as key precursors for the formation of plant- and microbial-derived C, respectively (Liang and Zhu, 2021). In the present study, the limited exogenous C inputs in the older peanut field, as evidenced by the reduced labile C resources in Table 2, created unfavorable conditions for the formation of plant-derived C. Concurrently, the limited C resources may create a ‘resource-poor’ environment for microbes, thereby hindering microbial growth (Table 3) and subsequently reducing the production of microbial residues (Hu et al., 2024). The positive correlations between microbial biomass, PC, and labile C pools as well as between microbial biomass and MRC, as demonstrated in PLS-PM analysis (Figure 4), corroborated these findings. Furthermore, the scarcity of C resources in the older peanut fields could, to some extent, drive microbes gain energy for their survival from the decomposition of pre-existing PC and MNC (Shao et al., 2021). This was partially corroborated by the findings presented in Table 6, which demonstrate a high degree of lignin’s degradation, indicated by the reduced S/V and C/V ratios observed in these fields. Overall, under the premise of insufficient SOC and labile C resources in the older peanut fields, the diminished formation of ‘new’ MRC and PC, coupled with the increased decomposition of ‘old’ MRC and PC, collectively contribute to reduced MRC and PC accumulation in these fields (Tables 5, 6).

Notably, it was observed that the MRC/SOC values were significantly elevated in the elder peanut fields compared to other treatments, whereas the PC/SOC values exhibited inverse age-dependent trends relative to the MRC/SOC patterns (Table 6), which means that long-term peanut cultivation enhances the microbial contribution to SOC, while diminishing the plant contribution to SOC. The reduced plant contribution to SOC could be easily explained by the sharply decline in PC in the older peanut fields, which were induced by diminished production of ‘new’ PC production and increased decomposition of ‘old’ PC. Notable, although microbial biomass and its residues (i.e., MRC) decreased sharply in the older fields (see Tables 3, 5), its contribution to SOC increased substantially. This seemingly contradictory finding can be mainly elucidated by the discrepancy in mineral protection intensity between MRC and PC in soil (Li et al., 2020). Recent studies posited that MRC, especially FRC, exhibits a greater resistance to microbial ‘attack’ than PC, owing to its enhanced mineral protection and inherent biochemical recalcitrance (Li et al., 2020; Niu et al., 2024). Consequently, the increased levels of reactive Fe oxides (i.e., Feo and Fep) in the older peanut fields may enhance mineral protection for MNC, which was confirmed by the positive correlation between Fe oxides and MNC (Figure 4). This partially accounts for the increased MRC/SOC ratios observed in these fields. Furthermore, Kallenbach et al. (2016) suggested that unfavorable soil conditions exert strong physiological stress on microorganisms, thereby accelerating their life cycle and resulting in a greater accumulation of microbial necromass per unit of living biomass. In the present study, the aggravated microbial (i.e., bacterial) physiological stress, attributed to the acidified conditions (pH 4.57–5.12) and C limited microenvironments in the older peanut fields, as indicated by the reduced Cy/Pre values, may facilitate microbial turnover and consequently resulted in relatively increased production of microbial residues. Collectively, the above-mentioned findings together explained the increased microbial contribution to SOC in the older peanut fields.

## 5 Conclusion

This study elucidates the coupled mineral–microbial patterns that regulate SOC dynamics along a 0–20-yr. peanut cultivation chronosequence. The main findings are as follows: (i) prolonged peanut cultivation (i.e., 10-and 20-yr.) adversely affected the accumulation of SOC resources (e.g., DOC, LFOC, MRC, and PC) and reduced SOC lability, while simultaneously increasing N levels and exacerbating soil acidification. (ii) initial stages of peanut cultivation (2- and 5-yr.) increased C and N supply levels, thereby fostering microbial growth and their residues (i.e., MRC) accumulation, but these positive effects are progressively negated by severe acidification and C limitation in the old peanut fields; (iii) the progressive soil acidification (with pH declining from 6.10 to 4.57) activates reactive Fe oxides (Feo and Fep), thereby shifting the Fe–C coupling from weak adsorption to strong complexation. This mineral-strengthening process preferentially preserves MRC via organo-mineral associations, consequently increasing the microbial contribution to SOC accumulation during peanut cultivation, despite the depletion of MRC. To address these soil problems (e.g., acidification, SOC/TN imbalance, and declining SOC levels) caused by long-term peanut cultivation, we propose the following interventions: (i) as the pH level decreased to 5.12 after a decade, accompanied by a significant reduction in microbial biomass, the implementation of various acidity-management strategies, e.g., appropriate liming, may become increasingly crucial as the duration of peanut cultivation extends; and (ii) incorporation of biochar or other high C:N organic amendments to replenish C resources and rectify the SOC/TN stoichiometric imbalance. These strategies are anticipated to enhance SOC stocks, improve soil aggregation, and ensure long-term productivity in intensive peanut cultivation systems in China.

## Data Availability

The original contributions presented in the study are included in the article/Supplementary material, further inquiries can be directed to the corresponding authors.
