# Supplementary material for: Long-term peanut cultivation alters soil organic C stocks and aggregate stability: insights from coupled iron oxide, microbial, and lignin phenol analyses
Source: Front Microbiol. 2026 Oct 9;17:1971411. doi: 10.3389/fmicb.2026.1971411 (PMC13690795; doi:10.3389/fmicb.2026.1971411)
Supplement: Supplementary file 1 [file Supplementary_file_1.docx]

**Table S1** Three microbial subgroups indicated by specific phospholipid fatty acid (PLFAs) biomarker in this study.

| Microbial subgroups | Specific PLFAs |
| --- | --- |
| Bacteria | a13:0, i13:0, a14:0, i14:0, a15:0, i15:0, a16:0, i16:0, a17:0, i17:0, a19:0, 15:1ω5c, 16:1ω7c, 16:1ω7c DMA, cy17:0, 17:1ω7c 10-methyl, 17:1ω8c, 18:1ω5c, 18:1ω7c, 18:1ω7c 10-methyl, cy19:0, 15:00, and 17:00 |
| Fungi | 16:1ω5c, 18:2ω6c, and 18:1ω9c |
| Actinomycetes | 10Me-16:0, 10Me-17:0, and 10Me-18:0 |

^#^ The sum of bacteria, fungi, and actinomycetes was quantified and represented as the total microbial biomass.

**Table S2** The detailed information of seven exo-enzymes in this study.

| Exo-enzymes | | Substrate | Abbreviation |
| --- | --- | --- | --- |
| Hydrolase | α-Glucosidase | 4-MUB-α-D-glucoside | αG |
|  | β-Glucosidase | 4-MUB-β-D-glucoside | βG |
|  | β-Cellobiosidase | 4-MUB-β-D-cellobioside | CBH |
|  | β-Xylosidase | 4-MUB-β-D-xyloside | βX |
|  | N-acetyl-glucosaminidase | 4-MUB-N-acetyl-β-D-glucosaminide | NAG |
| Oxidase | Phenol oxidase | *L*-DOPA | PHOs |
|  | Peroxidase | *L*-DOPA | PerX |

**Fig. S1.** Location of the experimental sites
